# Correction: Acute kidney injury – A frequent and serious complication after primary percutaneous coronary intervention in patients with ST-segment elevation myocardial infarction

**DOI:** 10.1371/journal.pone.0229346

**Published:** 2020-02-12

**Authors:** Abdellatif El-Ahmadi, Mujahed Sebastian Abassi, Hedvig Bille Andersson, Thomas Engstrøm, Peter Clemmensen, Steffen Helqvist, Erik Jørgensen, Henning Kelbæk, Frants Pedersen, Kari Saunamäki, Jacob Lønborg, Lene Holmvang

In Fig 1, the y-axis label is incorrect. The Fig 1 caption is also incorrect. Please see the correct Fig 1 and correct caption here.

**Fig 1:**
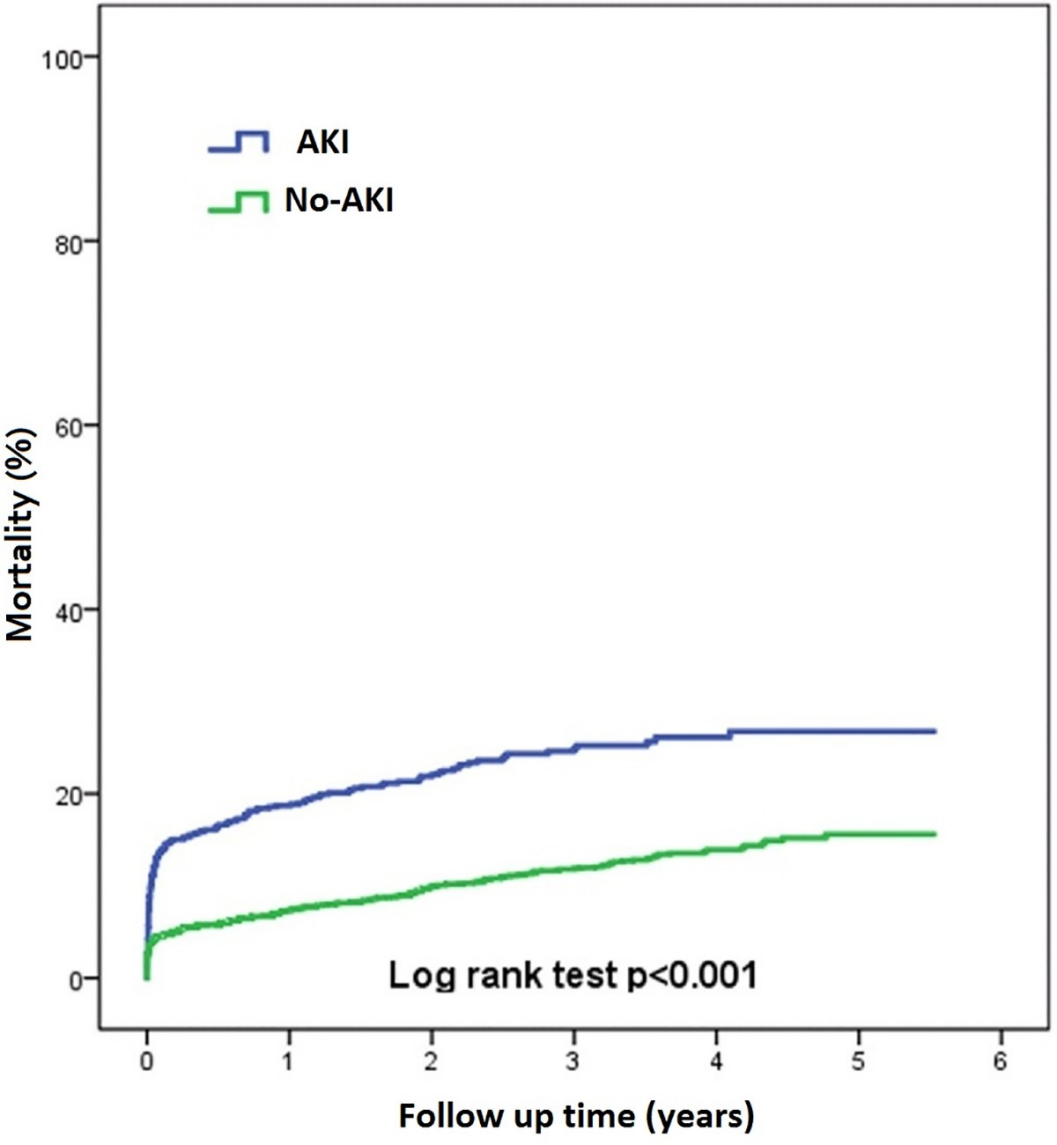
Mortality.
